# Circular RNA CHST15 Sponges miR-155-5p and miR-194-5p to Promote the Immune Escape of Lung Cancer Cells Mediated by PD-L1

**DOI:** 10.3389/fonc.2021.595609

**Published:** 2021-03-11

**Authors:** Jianru Yang, Yang Jia, Bing Wang, Shengrong Yang, Kun Du, Yujie Luo, Yunhe Li, Bing Zhu

**Affiliations:** ^1^ Department of Obstetrics and Gynecology, The Second Affiliated Hospital of Chongqing Medical University, Chongqing, China; ^2^ Department of Plastic Surgery, The Second Hospital of Hebei Medical University, Shijiazhuang, China; ^3^ Department of Thoracic and Cardiovascular Surgery, The Second Affiliated Hospital of Chongqing Medical University, Chongqing, China

**Keywords:** lung cancer, CircCHST15, miR-155-5p, miR-194-5p, PD-L1, immune escape

## Abstract

**Background:**

The effects of up-regulated CircCHST15 on lung cancer remained unclear. In this study, the role of CircCHST15 in lung cancer was investigated.

**Methods:**

Dual-luciferase reporter verified the bioinformatics prediction that CircCHST15 targeted miR-155-5p and miR-194-5p. The correlation between CircCHST15 and PD-L1 was analyzed by Pearson analysis. CCK-8 and colony formation was performed to determine the viability and proliferation of lung cancer cells. After the lung cancer (subcutaneous-xenotransplant) model was established in mice, the T cell subtype and related cytokines in mouse tumor tissues were detected by flow cytometry and ELISA. Moreover, the expressions of CircCHST15, miR-155-5p, miR-194-5p, immune-related, and proliferation-related factors of the lung cancer cells or mice tumor tissues were detected by immunohistochemistry, RT-qPCR, or Western blot.

**Results:**

CircCHST15 and PD-L1 were high-expressed in lung cancer, and the two was positively correlated. CircCHST15 targeted miR-155-5p and miR-194-5p, the later further targeted PD-L1. Lung cancer cell viability and proliferation were increased by miR-155-5p and inhibited by miR-194-5p. CircCHST15 located in the cytoplasm promoted tumor growth, down-regulated the expressions of miR-155-5p and miR-194-5p, and up-regulated the expressions of PD-L1, Ki-67, PCNA, CCL17, CCL22, IFN-γ, TNF-β, and IL-10. Also, CircCHST15 decreased the CD8^+^ cells in mouse blood and tumor, but increased the Tregs in mouse tumor. PD-L1 inhibitor showed an opposite effect to CircCHST15 on mouse tumors.

**Conclusion:**

CircCHST15 sponged miR-155-5p and miR-194-5p to promote the PD-L1-mediated immune escape of lung cancer cells.

## Introduction

Lung cancer originates from the bronchial mucosa, glands, or alveolar epithelium (1, 2), and is gradually becoming one of the most challenging cancers with high mortality (1, 3). According to the statistics, more than 1.8 million new cases of lung cancer and 1.6 million deaths occur annually (1, 3). As early-staged lung cancer is not easily detectable, more than 70–80% of lung cancer patients have already missed the optimal chance for surgery by the time of diagnosis (1, 3, 4). Chemotherapy is now a main treatment for patients with advanced lung cancer, but is accompanied with various adverse side effects (5). Hence, it is necessary to seek new strategies of treatment for patients with lung cancer.

The development of cancers is dependent not only on the characteristics of cancer cells, but also on the interaction of cancer cells with the immune system (5, 6). Studies showed that the microenvironment of tumor allow tumor cells to penetrate into lymphatic vessels and blood vessels, evade the immune surveillance of the body, resist the killing effect of antigen-specific T cells, thus realizing distant metastasis and growth (7). However, the progression and mechanism through which tumor cells evade immune surveillance have not been fully elucidated.

Circular RNAs (circRNAs), a category of non-coding RNAs, is featured by a covalently closed-loop structure and do not have the 5′ and 3′ ends normally contained in linear RNAs (8). In recent decades, evidence has increasingly proven that circRNAs play crucial parts in cancer development, including lung cancer (9–11). For example, CircZNF124 can promote the progression of non-small cell lung cancer cells by targeting mIR-337-3p (12); hsa_circ_0011780 inhibits the proliferation and metastasis of lung cancer (13); down-regulated CircABCB10 enhances the sensitivity of lung cancer cells to cisplatin (14); hsa_circ_0072083 could promote the effect of cisplatin on suppressing the growth of lung cancer (15). Furthermore, a recent study discovered that CircCHST15 (hsa_circ_0109320) is differentially expressed in gefitinib-treated lung cancer and might be a biomarker for the treatment of lung cancer in the future (16). However, the specific role of CircCHST15 in lung cancer still remained unknown. In addition, circRNAs function as spongers to miRNAs and modulate the progression of cancers, including the progression of immune escape (10). The miRNAs sponged by CircCHST15 should be further determined.

On the basis of the current backgrounds, this study focused on determining the specific role of CircCHST15 on lung cancer, especially in the progression of immune escape and the potential miRNAs sponged by CircCHST15 in lung cancer.

## Methods

### Ethics Statement

All the experiments involved in clinical samples were authorized by the Ethics Committee of the Second Affiliated Hospital of Chongqing Medical University (Z20190625H). All patients were informed with the use of their samples in this study, and all signed informed consent. All the animal experiments in this study were approved by the Committee of Experimental Animals of the Second Affiliated Hospital of Chongqing Medical University (Z20190821H).

### Clinical Tissues

Cancer tissues and adjacent normal tissues were harvested from 90 lung cancer patients who have received surgery in the Second Affiliated Hospital of Chongqing Medical University between December 2017 and May 2019. None of the patients had received chemotherapy or radiotherapy prior to the study. The major clinical features of the patients (**Table 1**), including age of patients, grade, tumor size, clinical stage, differentiation, and lymph node metastasis, were also collected from the Second Affiliated Hospital of Chongqing Medical University.

**Table 1:** Comparison of CircCHST15 expression level in peripheral blood of patients with different clinical pathology characteristics.

Group	n	Low (n = 45)	High (n = 45)	P value
Age (years)				
≤60	35	19	16	0.517
>60	55	26	29	
Grade				
T1	38	17	21	0.169
T2	35	16	19	
T3	17	12	5	
Tumor size				
<5 cm	48	27	21	0.205
>=5 cm	42	18	24	
Clinical stages				
I+II	56	36	20	0.001
III+IV	34	9	25	
Differentiation				
Well	31	19	12	0.299
Moderately	32	14	18	
Poorly	27	12	15	
Lymph node metastasis				
Yes	23	7	16	0.030
No	67	38	29	

### Cell Culture

Human normal bronchial epithelial cell 16HBE (CL-0249, Procell, Wuhan, China, https://www.procell.com.cn); human lung cells including H1299 (CRL-5803, ATCC), H23 (CRL-5800, ATCC), H1359 (CRL-5868, ATCC), H1435 (CRL-5870, ATCC), A549 (CCL-185, ATCC), H358 (CRL-5807, ATCC), and PC-9 (BNCC341852, BeNa Culture Collection, Beijing, China); and mouse lung cancer cell CMT-167 (Jennio Biotech, Guangzhou, China, http://www.jennio-bio.com/products_detail/&productId=534.html) were all grew in RPMI-1640 medium (21870076, Gibco, MA, USA) containing 10% FBS (10437010, Gibco) and cultured in a humid atmosphere with 5% CO_2_ at 37°C.

### RNase R Treatment

Briefly, 2.5 μg RNAs of H1395 and A549 cells were incubated with 10U RNase R (M1228, BioVision Incorporated, CA, USA) for 35 min at 37°C. The RNAs incubated with RNase R (RNase R+) or non-incubated RNase R (RNase R−) were used for the analysis of RT-qPCR.

### Transfection

Plasmids overexpressing CircCHST15 were ligated into pLV-cir vector, shRNA for CircCHST15, and negative control for shRNA were synthesized by Miaoling Biological (Wuhan, China, http://www.miaolingbio.com/). The pLV-cir vector used alone served as an overexpression negative control (NC). Mimics of miR-155-5p (miR10000646-1-5) and miR-194-5p (miR10000460-1-5), and negative control of mimic (MC; miR01102-1-1) were ordered from RIBOBIO (Guangzhou, China). Before the transfection, the H1395 (1.0 × 10^6^) and A549 (1.0 × 10^6^) cells were separately seeded into six-well plates, with each well containing 2 ml RPMI-1640 medium with 10% FBS. After the cell reached 80% confluence, the medium supplemented with FBS was replaced with an equal volume of RPMI-1640 medium without FBS. Then 2 μg CircCHST15, shCircCHST15, and mimics were respectively mixed with 100 μl Opti-MEM (31985070, Gibco). Three μl lipofectamine 2000 (11668-019, Invitrogen, MA, USA) was also diluted with 100 μl Opti-MEM. After incubation for 20 min at room temperature, the diluted lipofectamine 2000 with mimics or plasmids were added into each well of the six-well plates to incubate with the cells for 48 h.

### Dual-Luciferase Reporter Assay

Before the experiment, the wide-type sequence of CircCHST15 (CircCHST15-WT) carrying the binding site of miR-155-5p (5’-AGGACTATCCAAGCATTAA-3’), CircCHST15-WT carrying the binding site of miR-194-5p (5’-ATTGCTGCATACAGCTGTTACC-3’), the mutant sequence of CircCHST15 (CircCHST15-MUT; 5’-ATTGCTGCATACAAACTACTAC-3’), the WT sequence of PD-L1 (PD-L1-WT) carrying the binding site of miR-155-5p (5’-GTCACTTTTTGUACCTGCATTAA-3’), PD-L1-WT carrying the binding site of miR-194-5p (5’-GATATATTGTAGTAGATGTTACA-3’), and the mutant sequence of PD-L1 (PD-L1-MUT; 5’-GATCTGTACTCACAAAACCCGUA-3’) were respectively inserted into the pGL3-basic vectors (VT1554, YouBio, Hunan, China). Furthermore, the H1395 (3.0 × 10^4^) cells were added into 48-well plates, with each well containing 300 μl RPMI-1640 medium with 10% FBS. After the cell reached 80% confluence, the medium with FBS was replaced with an equal volume of RPMI-1640 medium without FBS. Then the H1395 cells were co-transfected with the vectors and miR-155-5p mimic, miR-194-5p mimic, or MC for 48 h, and then performed with dual-luciferase reporter experiment (ab228530, Abcam, CA, USA). The absorbance of the 48-well plates was detected by SpectraMax reader (Molecular Devices, Shanghai, China) for the activity of luciferase.

### Biotinylated RNA Pull-Down Assays

Briefly, the H1395 and A549 cells were first transfected with overexpression CircCHST15 plasmids for the up-regulation of the expression of CircCHST15 in the cells. Then, the two cells were further transfected with biotinylated miR-155-5p-WT, miR-155-5p-MUT, miR-194-5p-WT, or miR-194-5p-MUT. Next, the H1395 and A549 cells were lysed, sonicated, and then incubated with C-1 magnetic beads (C37488, Invitrogen) overnight at 4°C. The second day, after probe-coated beads were eluted, RT-qPCR was performed on the rest samples to ensure that CircCHST15 was pulled down by miR-155-5p-WT and miR-194-5p-WT.

### CCK-8 Assay

After the transfection, the H1395 (1.0 × 10^4^) and A549 (1.0 × 10^4^) cells were respectively added into each well of 96-well plate supplemented with 100 μl RPMI-1640 medium with 10% FBS. After the cell growth for 24 and 48 h, 10 μl of CCK-8 (A56097, OKA, Beijing, China) was further added into each well of the plate for further incubation for 4 h. Then the 96-well plate was inserted into the Imark microplate reader (Bio-Rad, CA, USA) to measure the absorbance under 570 nm.

### Colony Formation Assay

After the transfection, the H1395 (1.0 × 10^3^) and A549 (1.0 × 10^3^) cells were transferred into a six-well plate, with each well containing 2 ml RPMI-1640 medium with 10% FBS. During the 14-day cell culture, the medium was refreshed every 2 days. Subsequently, after the medium in the plate was discarded, the cells in each well were fixed with 4% paraformaldehyde prepared with 100% paraformaldehyde (ALFA11313, OKA) and PBS (10010049, Gibco). After that, the cells were then dyed with purple crystal (D10739, OKA) for 10 min at room temperature. Finally, the stained cells in the well were observed under a DM4M optical microscope (Solms, Germany), and quantified by Image J 1.8.0 software.

### Animal Study

Thirty-three male BALB/c mice (age: 4 weeks old; weight: 18–20 g) were purchased from Cavens (Changzhou, China, http://www.cavens.com.cn/). All the mice were kept in an SPF environment with 12-h light/12-h dark cycle and provided with free diet. The mice were acclimated for 5 days before the experiment. The animal experiments in this study consisted of two sections.

In the first section, 12 mice were divided into four groups as follows: the NC group (n = 3), the Control group (n = 3), the mock group (n = 3), and the shCircCHST15 group (n = 3). Before the operation, the CMT167 cells were transfected with lentivirus without any vector (NC lentivirus) or with lentivirus containing shCircCHST15 vector (shCircCHST15 lentivirus). The vectors were synthesized by Genepharma (Shanghai, China). After the transfection, 2.0 × 10^7^ normally cultured CMT167 (for the Control group) transfected with NC lentivirus (for the mock group) or shCircCHST15 lentivirus (for the shCircCHST15 group) were injected into the right forelimb subcutaneous armpit of the mice in the Control group, the mock group, and the shCircCHST15 group, respectively. The mice in the NC group were not performed with any injection. Then the mice were normally raised, and 4 days after the injection, when a tumor of 4–5 mm under the armpit of the mouse was touchable, this indicated that lung cancer mouse model was established successfully. Twenty-eight days after the injection, all the mice were sacrificed by cervical dislocation under anesthesia with 0.2% phenobarbital sodium (B005, Nanjing Jiancheng Bio, Jiangsu, China). Then the tumor and blood of all the mice were collected for later use.

In the second section, 21 mice were divided into seven groups: the NC group (n = 3), the Control group (n = 3), the Anti-PD-L1 group (n = 3), the mock group (n = 3), the CircCHST15 group (n = 3), the Anti-PD-L1+CircCHST15 group (n = 3), and the shCircCHST15 group (n = 3). Before the operation, the CMT167 cells were transfected with NC lentivirus, CircCHST15 lentivirus, or shCircCHST15 lentivirus. After the transfection, 2.0 × 10^7^ normally cultured CMT167 cells (for the Control group and the Anti-PD-L1 group) or those transfected with NC lentivirus (for the mock group), CircCHST15 lentivirus (for the CircCHST15 group and the Anti-PD-L1+CircCHST15 group), or shCircCHST15 lentivirus (for the shCircCHST15 group) were respectively injected into the right forelimb subcutaneous armpit of mice. The mice in the NC group were normally raised, and intraperitoneally injected with 300 μg saline on days 8, 10, 12. On days 8, 10, and 12 after the injection, the mice in the NC group, the Control group, the mock group, the CircCHST15 group, and the shcircCHST15 group were intraperitoneally injected with normal saline (300 μg/mouse; R21479, OKA), while those in the Anti-PD-L1 group and the Anti-PD-L1+CircCHST15 group were intraperitoneally injected with PD-L1 inhibitor (300 μg/mouse; P872738, Macklin, Shanghai, China). Twenty-eight days after injection, all the mice were sacrificed by cervical dislocation under anesthesia with 0.2% phenobarbital sodium. Then the tumor and blood of all the mice were collected for later use.

### Flow Cytometry

The proportion of CD4^+^ and CD8^+^ cells in peripheral blood and tumor supernatant of the mice was detected by flow cytometry. Specifically, after incubation with the CD4 antibody labeled with FITC (ab59474, Abcam) for 1 h, the samples were further incubated with CD8 antibody labeled with PE (ab59474, Abcam) for 1 h. After that, the samples were further incubated with CD3 antibody conjugated with violetFluor 450 (38527, CST, MA, USA) and CD25 antibody conjugated with PE-Cy7 (43212, CST) for 1 h. Then the percentages of CD4^+^, CD8^+^, CD3^+^, CD25^+^ cells in the samples were quantified by flow cytometer system (FACS-LSR II; Becton-Dickinson, NJ, USA). Finally, the percentage of Tregs^+^ in the samples were further measured based on data of the percentage of CD4^+^ and CD8^+^ cells according to the formula: Tregs^+^ (%) = [(CD3^+^ + CD4^+^ + CD25^+^ + total cell number)/CD3^+^ + total cells number] × 100%.

### ELISA

The mouse ELISA kits of IL-10 (ml037873), TNF-β (ml002098), and IFN-γ (ml002277) were commercially ordered from Enzyme-linked Biotechnology (Shanghai, China). In brief, tumor tissue supernatant (50 μl) was added into several wells of the 96-well plate provided in the ELISA kit, meanwhile, 50 μl different doses of standard samples from the ELISA kit were added into the rest wells. Soon after, 50 µl target antibody was transferred into the well containing the test sample or standard sample. After 1 h of incubation and washing twice, the well was then added with 80 μl HRP and incubated for 30 min at 37°C. An equal volume of solution A and solution B were mixed together to a total volume of 100 μl and added into the well for 20-min incubation. After stop buffer (50 µl) was added into the well, the absorbance of the 96-well plate was read by the Imark microplate reader (Bio-Rad, CA, USA) under 450 nm.

### Immunohistochemistry

The tumor tissues of mice were fixed with 4% paraformaldehyde and made transparent by rxylene (ALFL13317, OKA). Next, the tissues were embedded into paraffin (A56132, OKA), sectioned into 4 μm, and dewaxed. After soaking in antigen repair buffer (R20902, OKA) for 8 min, the tissues were further incubated with the antibody of PD-L1 (13684, CST) overnight at 4°C, soaked in goat-anti-rabbit antibody (ab205718, Abcam) for 1 h, and further dyed with DBA (SFQ004, 4A Biotech, Beijing, China). After washing the tissues under tap water, the tissues were then dyed with hematoxylin (D10519, OKA) for 4 min and further made transparent by xylene. Finally, the expression image of PD-L1 in the tumor tissue was observed under a DMLA full automatic microscope (Leica, Solms, Germany).

### Western Blot

The total protein from H1395 cells, A549 cells, and the mouse tumor tissues were extracted for determining the expressions of some key proteins. In short, after the extraction of total protein from samples using NP-40 (R21234, OKA), the concentration of the total protein was determined by the BCA Protein Assay Kit (A53226, Thermo Scientific, MA, USA). Then the protein was denatured by mixing with loading buffer (D10575, OKA) and heating at 100°C for 5 min. After seeding the protein into 10% SDS-PAGE gel (CW0022, CWBIO, Beijing, China), the denatured protein (25 μl) was electrophoresed (100 V) in the gel for 1.5 h at 4°C and then transferred to the PVDF membrane (JKA40001, OKA). Blocking buffer (CW2134, CWBIO) was applied to block the membrane. The following primary antibodies were bound with the protein on the membrane overnight at 4°C: CCL22 (1:1,000, 10 kDa, ab124768, Abcam), PD-L1 (1:2,000, 50 kDa, ab213480, Abcam), Ki67 (1:2,000, 359 kDa, ab16667, Abcam), CCL17 (1:1,500, 11 kDa, ab182793, Abcam), PCNA (1:2,000, 29 kDa, ab92552, Abcam), and GAPDH (1:5,000, 36kDa, ab8245, Abcam). After that, the mouse second antibody (1:10,000, ab205719, Abcam) was bound with the protein for another 2 h. Finally, the protein signal on the membrane was measured by the Image Lab 3.0 Software (Bio-Ras, CA, USA).

### RNA Extraction

Total RNA from clinical tissues, cultured cells, and mouse tumor tissues were used for determining the expressions of mRNAs and circRNAs. In brief, the samples were lysed by TRIzol (15596, Invitrogen), mixed with chloroform (A53235, OKA) and centrifuged (14,000 × *g*) for 30 min for the collection of supernatant. The supernatant was then incubated with an equal volume of isopropanol (E15794, OKA) for 15 min. After centrifuging (14,000 × g) supernatant for 5 min, the total RNA sediments were obtained.

Cytoplasm and nuclear RNA from H1395 and A549 cells were detected for the expression location of CircCHST15. The reagents used for the extraction of cytoplasm and nuclear RNA were the kit (37400, NORGEN BIOTEK, Thorold, Canada) specially used for the cytoplasm and nuclear RNA extraction. In short, after the cells were lysed by Buffer J and centrifuged (14,000 × g) for 15 min, the supernatant (cytoplasm RNA) and sediment (nuclear RNA) were respectively collected. The extraction of cytoplasm and nuclear RNA was basically the same, briefly, after incubation with Buffer SK (200 μl) for 10 s, the supernatant and sediment were respectively mixed with 200 μl ethanol (ALF045844, OKA), and the mixtures were centrifuged (3500 × *g*) for 2 min for harvesting the cytoplasm and nuclear RNA.

Cytoplasm and nuclear RNA from H1395 and A549 cells were detected for the expressions of miRNAs. The reagents used for the extraction of miRNA were the kit (DP501, TianGEN, Beijing, China) specially used for miRNA extraction. To be brief, after the cells were lysed by lysis buffer, the cells were mixed with chloroform and centrifuged (13,400 × *g*) for 30 min. Then the miRNA sediment was washed by 75% ethanol (A171299, Aladdin, Shanghai, China) once and collected for later use.

### RT-qPCR

The total RNAs and miRNAs were isolated from all the samples and reverse-transcribed into cDNAs using Omniscript RT Kit (205111, Qiagen, Dusseldorf, Germany, https://www.qiagen.com/cn/). Then the cDNA was amplified using the qPCR Qupermix Kit (AQ601-01, TransGen, Beijing, China) in the QuantStudio6 system (Applied Biosystems, CA, USA). The reaction conditions were as follows: at 94°C (30 s) for 1 cycle, at 94°C (30 s) for another 40 cycles, at 60°C (30 s) for 40 cycles. Finally, the expression of RNA was quantified by 2^−△△CT^ method. The primers for cDNAs amplification are shown in **Table 2**.

**Table 2:** RT-qPCR primers.

Target gene	Forward primers, 5’-3’	Reverse primers, 5’-3’
Human CircCHST15 Mouse CircCHST15 Human CHST15	GTAAGGTGTGAAGGCTGGCT CTACGAGACCGCTACCCTGT AATTCTGTTTCGTGTGGACAGT	TGGCAAAGCCTTTAAAGAGTCC TGAGCTTGGCTTCTGGTTGA CCCCAGTTTTCATTGCCCTCA
Human miR-155-5p Mouse miR-155-5p	ATCGTGATAGGGGTTGTCGT GGGTGTCGTATCCAGTGCAA	GTATCCAGTGCGTGTCGTGG GTCGTATCCAGTGCGTGTCG
Human miR-194-5p Mouse miR-194-5p	TGGAGTCGTATCCAGTGCAA TGGAGTCGTATCCAGTGCAA	GTCGTATCCAGTGCGTGTCG GTCGTATCCAGTGCGTGTCG
Human miR-671-5p Human miR-650 Human miR-3612 Human PD-L1 Human U6 Mouse U6 Human GAPDH Mouse GAPDH	GGAGGTCGTATCCAGTGCAA ACACAGTCGTATCCAGTGCAA TGGAGTCGTATCCAGTGCAA TGGCATTTGCTGAACGCATTT CTCGCTTCGGCAGCACA CAGCGCAGAATCACCCCAT GGAGCGAGATCCCTCCAAAAT AGGTCGGTGTGAACGGATTTG	GTCGTATCCAGTGCGTGTCG GTATCCAGTGCGTGTCGTGG GTCGTATCCAGTGCGTGTCG TGCAGCCAGGTCTAATTGTTTT AACGCTTCACGAATTTGCGT TTGATGTTCATCCAGTTGTCACA GGCTGTTGTCATACTTCTCATGG GGGGTCGTTGATGGCAACA

### Bioinformatics Analysis

The potential target miRNAs of CircCHST15 were predicted by TargetScan 7.2 (http://www.targetscan.org/vert_72/) and StarBase (http://starbase.sysu.edu.cn/index.php). Venn diagram of the potential target miRNAs of CircCHST15 was generated by Venny 2.1.0 (https://bioinfogp.cnb.csic.es/tools/venny/). The binding sites between CircCHST15 with miR-155-5p and between CircCHST15 with miR-194-5p, and the binding sites between PD-L1 with miR-155-5p and between PD-L1 with miR-194-5p were predicted in starBase.

### Statistical Analysis

Student’s t-test and one-way ANOVA were applied to analyze all the data in the current study. LSD and Dunnet’s served as *post-hoc* tests. The correlation of CircCHST15 expression with PD-L1 expression in the clinical samples was analyzed by Pearson correlation analysis. All the analyses in this study were performed in SPSS 19.0 software. Statistical data were finally presented as Mean ± SD. Statistically significance was defined when *P* < 0.05.

## Results

### CircCHST15 Was High-Expressed in Lung Cancer Tissues and Cells, and CircCHST15 Was Mainly Expressed in the Cytoplasm

**Figure 1:**
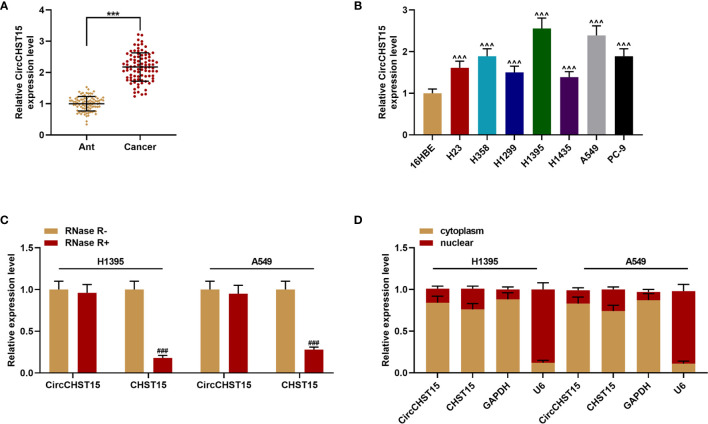
CircCHST15 expression was up-regulated in lung cancer and the gene was mainly expressed in the cytoplasm. **(A)** The expression of CircCHST15 in lung cancer tissues and adjacent normal tissues was detected by RT-qPCR, GAPDH was used as an internal control (^***^ *P* < 0.001, *vs.* Ant). **(B)** The expression of CircCHST15 in lung cancer cells and normal bronchial epithelial cells was detected by RT-qPCR, GAPDH was used as an internal control (^^^^^ *P* < 0.001, *vs.* 16HBE). **(C)** The expressions of CircCHST15 and CHST15 were detected by RT-qPCR in H1395 and A549 cells in which RNAs were treated with or without RNase R, GAPDH was used as an internal control (^###^ *P* < 0.001, *vs.* RNase R−). **(D)** Relative CircCHST15 expression in the cell cytoplasm or nucleus of H1395 and A549 cells was determined by RT-qPCR, GAPDH was used as the cytoplasmic internal control, and U6 was used as the nuclear internal control. (Ant, adjacent normal tissues).

The major clinical features of the collected tissues were analyzed, and we found that the clinical stage of the patients with the high-expressed CircCHST15 was more advanced than those with low-expressed CircCHST15 (*P* < 0.001, **Table 1**), also the lymph node metastasis of patients with the higher level of CircCHST15 was severer than that in patients with low-expressed CircCHST15 (*P* < 0.001, **Table 1**). To determine the role of CircCHST15 in lung cancer, the expression of CircCHST15 in lung cancer was analyzed. As compared with the adjacent normal tissues (the ANT group) and normal bronchial epithelial cells (16HBE), the expression of CircCHST15 was up-regulated both in cancer tissues (*P* < 0.001, **Figure 1A**) and cancer cells (*P* < 0.001, **Figure 1B**). The H1395 and A549 cells were used for the *in vitro* study because the expression of CircCHST15 in the two cells was relatively higher than in other cancer cells. Mechanically, the loop structure of CircCHST15 was verified by treating the RNA isolated from the two cells with RNase R (**Figure 1C**), and the results demonstrated that the expression of CHST15 was significantly down-regulated (*P* < 0.001), while no obvious difference was found in CircCHST15 expression after treatment with RNase R (RNase R+) in comparison with the RNase R− group. Also, the expression of CircCHST15 in the cytoplasm of the two cells was apparently higher than that in the nucleus (**Figure 1D**), suggesting that CircCHST15 was mainly expressed in the cytoplasm.

### ShCircCHST15 Inhibited Tumor Growth, the Levels of IFN-γ, TNF-β, IL-10, PD-L1, and Regulated the T Cell Subtype in Lung Cancer Model Mice

**Figure 2:**
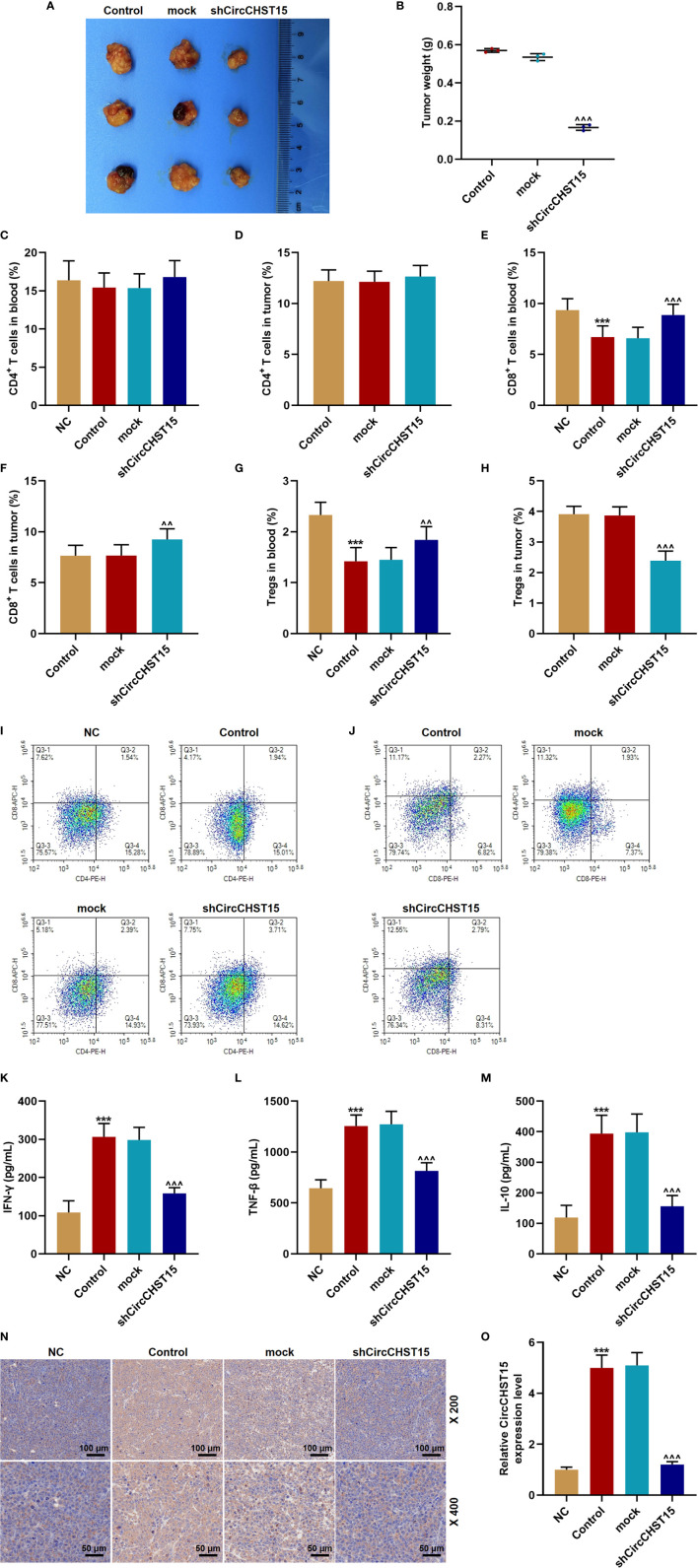
ShCircCHST15 inhibited tumor growth, the expressions of IFN-γ, TNF-β, IL-10, PD-L1, and regulated the T cell subtype in lung cancer model in mice. **(A)** The solid tumors were observed. **(B)** The weight of a solid tumor was calculated. **(C, D)** The level of CD4^+^ T cells in the blood **(C)** and tumor supernatant **(D)** of mice was quantified by the flow cytometer. **(E, F)** The level of CD8^+^ T cells in the blood **(E)** and tumor supernatant **(F)** of mice was quantified by the flow cytometer. **(G, H)** The level of Tregs in the blood **(G)** and tumor supernatant **(H)** of the mice was calculated based on the formula: Tregs^+^ (%) = [(CD3^+^ + CD4^+^ + CD25^+^ + total cell number)/CD3^+^ + total cells number] × 100%. **(I, J)** The proportion of immune cell subsets in peripheral blood and tumor was measured by flow cytometry. **(K–M)** The levels of IFN-γ **(K)**, TNF-β **(L)**, and IL-10 **(M)** in the blood of the mice were detected by ELISA. **(N)** The expression of PD-L1 in the mouse tumor tissues was detected by immunohistochemical. **(O)** The expression of CircCHST15 in the mouse tumor tissues was detected by RT-qPCR, GAPDH was used as an internal control. (***P < 0.001, *vs.* NC; ^^^^ *P* < 0.01, ^^^^^ *P* < 0.001, *vs.* mock).

In the lung cancer (subcutaneous-xenotransplant) model mice and their tumor tissues (**Figure 2A**), we found that siCircCHST15 obviously reduced the tumor weight in comparison with the mock group (*P* < 0.001, **Figure 2B**). Then the T cell subtype in the blood of mice and the tumor supernatant was analyzed, and the data revealed that the percentage of CD4^+^ T cells in the blood and tumor had no changes among all the groups (**Figures 2C, D, I, J**), while the percentage of CD8^+^ T cells in the blood of the control mice was reduced compared with the NC group (*P* < 0.001, **Figures 2E, I, J**) and increased by shCircCHST15 both in the blood (**Figures 2E, I, J**) and tumor supernatant (**Figures 2F, I, J**) when compared with the mock group (*P* < 0.01). The Tregs in the blood of control mice was reduced as compared with the NC group (*P* < 0.001, **Figure 2G**) and increased by shCircCHST15 both in the blood (**Figure 2G**) and tumor supernatant (**Figure 2H**) as compared with the mock group (*P* < 0.01). These results demonstrated that the effect of CircCHST15 on tumor growth was mediated by regulating the activation of CD8^+^ T cells in the blood and tumor. Therefore, the expressions of key factors (IFN-γ, TNF-β, IL-10) specific to CD8^+^ T were determined. As shown in **Figures 2K–M**, the expressions of IFN-γ, TNF-β, and IL-10 were up-regulated in the blood of the control mice when compared with the NC group (*P* < 0.001), but down-regulated by shCircCHST15 when compared with the mock group (*P* < 0.001). As PD-L1 regulated the immune escape in many cancers, we also detected its expression in the tumor tissues (**Figure 2N**), and discovered that the PD-L1 expression in the control and mock mice was down-regulated and it could be up-regulated by shCircCHST15. Additionally, we also found that the expression of CircCHST15 was significantly up-regulated in the control mice when compared with the NC group (*P* < 0.001, **Figure 2O**), but down-regulated by shCircCHST15 when compared with the mock group (*P* < 0.001, **Figure 2O**).

### CircCHST15 Regulated PD-L1 Expression in Lung Cancer Cells and It Was Positively Correlated to the Expression of PD-L1

**Figure 3:**
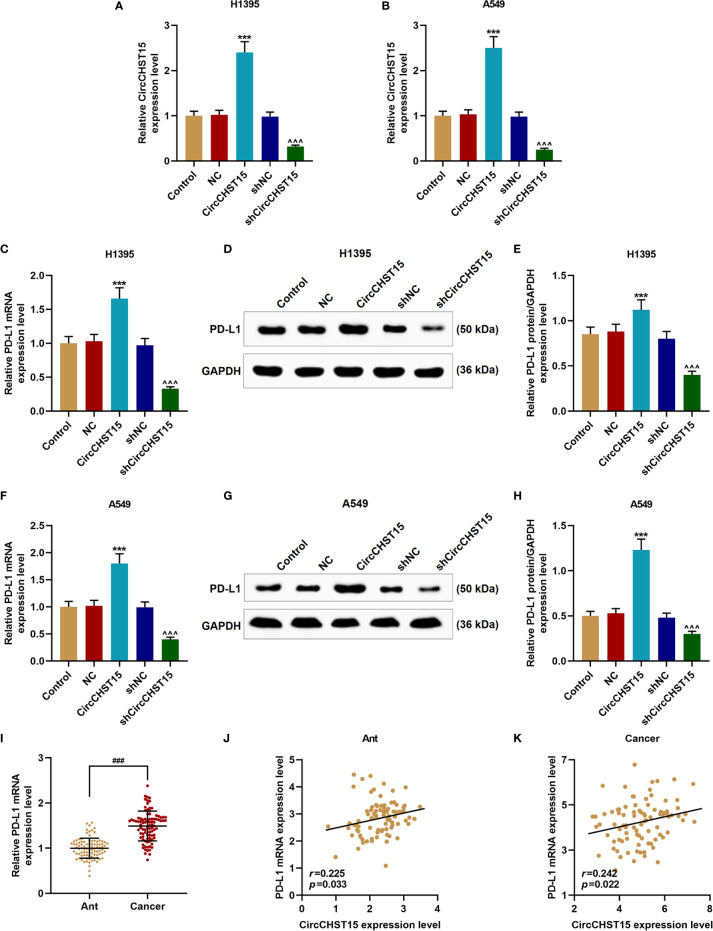
CircCHST15 regulated PD-L1 expression in lung cancer cells and it was positively correlated to the expression of PD-L1. **(A, B)** The transfection efficiencies of CircCHST15 and shCircCHST15 in H1395 **(A)** and A549 **(B)** cells were determined by RT-qPCR, GAPDH was used as an internal control. **(C, F)** The gene expressions of PD-L1 in H1395 **(C)** and A549 **(F)** cells were determined by RT-qPCR, GAPDH was used as an internal control. **(D, E, G, H)** The protein expression of PD-L1 in H1395 **(D, E)** and A549 **(G, H)** cells was determined by western blot, GAPDH was used as an internal control. **(I)** The expression of PD-L1 in Ant and lung cancer tissues was determined by RT-qPCR, GAPDH was used as an internal control. **(J, K)** The correlation between the expressions of CircCHST15 and PD-L1 in Ant **(J)** and lung cancer tissues **(K)** was analyzed by Pearson correlation analysis. (^***^ *P* < 0.001, *vs.* NC; ^^^^^ *P* < 0.001, *vs.* shNC). (Ant, adjacent normal tissues).

Then the expression of PD-L1 in the H1395 and A549 cells overexpressed with CircCHST15 or knocked down of CircCHST15 (**Figures 3A, B**) was measured to examine whether PD-L1 was regulated by CircCHST15. It could be observed that the gene and protein expressions of PD-L1 in H1395 cells (**Figures 3C–E**) and A549 cells (**Figures 3F–H**) were up-regulated by CircCHST15 (*P* < 0.001) and down-regulated by shCircCHST15 (*P* < 0.001) when compared with the NC group and shNC group, respectively. As we found that the expression of PD-L1 was high-expressed in lung cancer tissues in comparison with the Ant group (*P* < 0.001, **Figure 3I**). The correlation between the expressions of CircCHST15 and PD-L1 was analyzed. As shown in **Figure 3J** and **Figure 3K**, a positive correlation was identified between CircCHST15 and PD-L1 both in adjacent normal tissues and in lung cancer tissues.

### CircCHST15 Sponged miR-155-5p and miR-194-5p, and the Two miRNAs Targeted PD-L1

**Figure 4:**
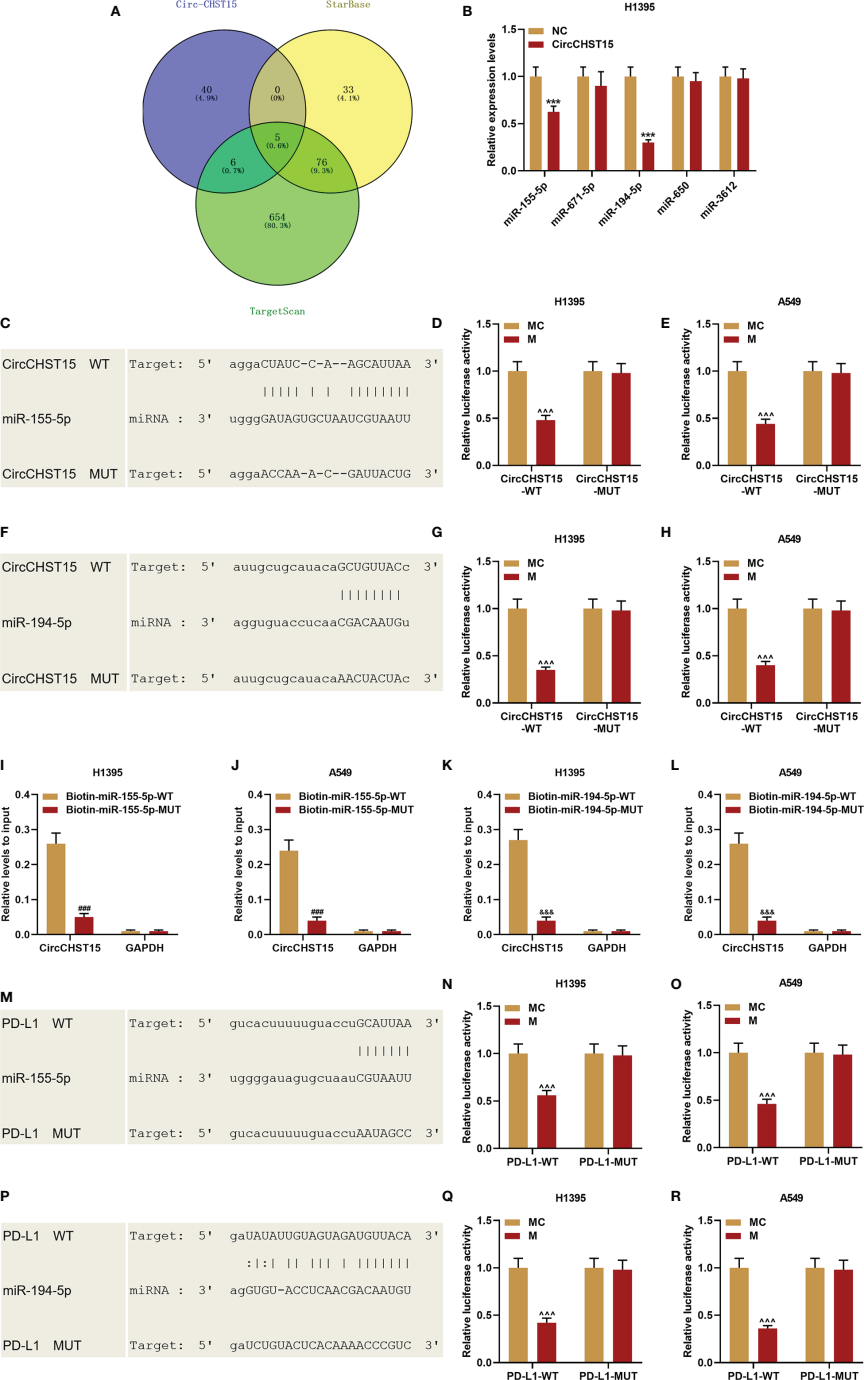
CircCHST15 sponged miR-155-5p and miR-194-5p, and the two miRNAs further targeted PD-L1. **(A)** Five miRNAs that might be sponged by CircCHST15 were predicted by bioinformatics. **(B)** The expressions of the five miRNAs (miR-155-5p, miR-671-5p, miR-194-5p, miR-650, and miR-3612) in H1395 cells transfected with CircCHST15 were determined by RT-qPCR, U6 was used as an internal control. **(C, F)** The binding sites between CircCHST15 with miR-155-5p **(C)** and between CircCHST15 with miR-194-5p **(F)** were predicted by bioinformatics. **(D, E, G, H)** Dual-luciferase reporter assays validated that CircCHST15 sponged miR-155-5p and miR-194-5p in H1395 and A549 cells. **(I–L)** Biotinylated RNA pull-down assays validated that CircCHST15 sponged miR-155-5p and miR-194-5p in H1395 and A549 cells. **(M–P)** The binding sites between PD-L1 with miR-155-5p **(M)** and between PD-L1 with miR-194-5p **(P)** were predicted by bioinformatics. **(N, O, Q, R)** Dual-luciferase reporter assays validated that PD-L1 was targeted by miR-155-5p and miR-194-5p in H1395 and A549 cells. (^***^ *P* < 0.001, *vs.* NC; ^^^^^ *P* < 0.001, *vs.* MC; ^###^ *P* < 0.001, *vs.* Biotin-miR-155-5p-WT; ^&&&^ *P* < 0.001, *vs.* Biotin-miR-194-5p-WT).

Bioinformatics predicted five miRNAs targeted by CircCHST15 (**Figure 4A**), among the five miRNAs, the expressions of miR-155-5p and miR-194-5p were down-regulated by CircCHST15 (**Figure 4B**). The binding sites between CircCHST15 with miR-155-5p (**Figure 4C**) and between CircCHST15 with miR-194-5p (**Figure 4F**) were also predicted and verified by dual-luciferase reporter assays. We found that the luciferase activities of H1395 cells (**Figures 4D, E**) and A549 cells (**Figures 4G, H**) co-transfected with CircCHST15-WT and mimic of miR-155-5p or miR-194-5p were reduced, however, no difference was found in the cells co-transfected with CircCHST15-MUT and mimic of miR-155-5p or miR-194-5p. In addition, the results of dual-luciferase reporter assays were further confirmed by biotinylated RNA pull-down assays (**Figures 4I–L**), the results of which also proved that the CircCHST15 sponged miR-155-5p and miR-194-5p. To determine whether miR-155-5p and miR-194-5p regulated PD-L1, the binding sites between PD-L1 with miR-155-5p (**Figure 4M**) and between PD-L1 with miR-194-5p (**Figure 4P**) were predicted by bioinformatics, and the target relationships were further verified by the results of dual-luciferase reporter assays (**Figures 4N, O, Q, R**).

### The Effects of CircCHST15 on the Expressions of miR-155-5p, miR-194-5p, and PD-L1 Were Reversed by miR-155-5p Mimic and miR-194-5p Mimic

**Figure 5:**
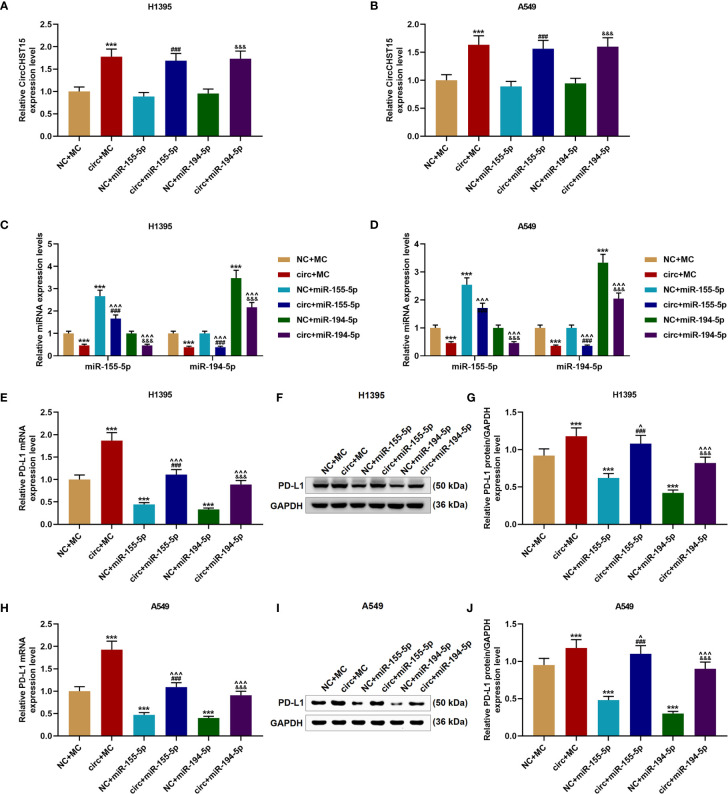
The effects of CircCHST15 on the expressions of miR-155-5p, miR-194-5p, and PD-L1 were reversed by miR-155-5p mimic and miR-194-5p mimic. **(A, B)** The expression of CircCHST15 in H1395 cells **(A)** and A549 cells **(B)** after transfection was determined by RT-qPCR, GAPDH was used as an internal control. **(C, D)** The expressions of miR-155-5p and miR-194-5p in H1395 cells **(C)** and A549 cells **(D)** after transfection were determined by RT-qPCR, U6 was used as an internal control. **(E, H)** The gene expressions of PD-L1 in H1395 cells **(E)** and A549 cells **(H)** after transfection were determined by RT-qPCR, GAPDH was used as an internal control. **(F, G, I, J)** The protein expressions of PD-L1 in H1395 cells **(F, G)** and A549 cells **(I, J)** after transfection were determined by western blot, GAPDH was used as an internal control. (^***^ *P* < 0.001, *vs.* NC+MC; ^^^ *P* < 0.05, ^^^^^ *P* < 0.001, *vs.* circ+MC; ^###^ *P* < 0.001, *vs.* NC+miR-155-5p; ^&&&^ *P* < 0.001, *vs.* NC+miR-194-5p).

We then proved that miR-155-5p and miR-194-5p did not affect the expression of CircCHST15 in the H1395 cells (**Figure 5A**) or A549 cells (**Figure 5B**), but their expressions in the H1395 cells (**Figure 5C**) and A549 cells (**Figure 5D**) were down-regulated by CircCHST15 when compared with the NC+MC group (*P* < 0.001). After the co-transfection with the CircCHST15 and mimic (the circ+miR-155-5p group and the circ+miR-194-5p group), the expressions of miR-155-5p and miR-194-5p were up-regulated by mimics of miR-155-5p and miR-194-5p when compared with the circ+MC group (*P* < 0.001). In addition, the gene and protein expressions of PD-L1 in H1395 cells (**Figures 5E–G**) and A549 cells (**Figures 5H–J**) were up-regulated by CircCHST15 (*P* < 0.001) but down-regulated by miR-155-5p mimic (*P* < 0.001) and miR-194-5p mimic (*P* < 0.001) when compared with MC+NC group. However, in the circ+miR-155-5p group and the circ+miR-194-5p group, the expression of PD-L1 promoted by CircCHST15 was down-regulated by miR-155-5p mimic (*P* < 0.05) and miR-194-5p mimic (*P* < 0.001) when compared with the circ+MC group. These results indicated that the inhibitory effects of CircCHST15 on the expressions of miR-155-5p and miR-194-5p as well as the effect of CircCHST15 on promoting the expression of PD-L1 were reversed by miR-155-5p mimic and miR-194-5p mimic.

### The Proliferation of H1395 and A549 Cells Were Increased by miR-155-5p Mimic But Inhibited by miR-194-5p Mimic

**Figure 6:**
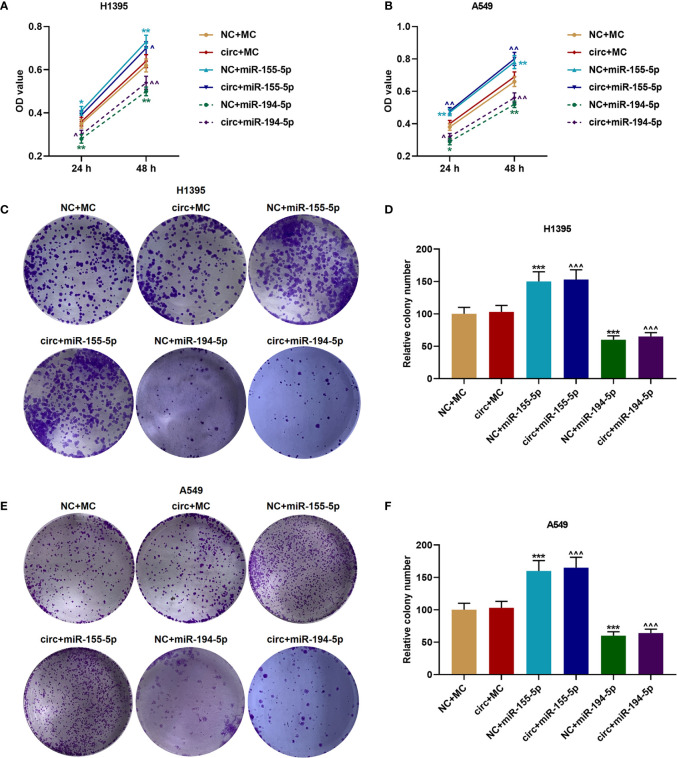
The proliferation of H1395 and A549 cells was enhanced by miR-155-5p mimic and inhibited miR-194-5p mimic. **(A, B)** The viabilities of H1395 cells **(A)** and A549 cells **(B)** after transfection were determined by CCK-8 assays. **(C, D)** The proliferation of H1395 cells after transfection was determined by colony formation assays. **(E, F)** The proliferation of A549 cells after transfection was determined by colony formation assays. (^*^ *P* < 0.05, ^**^ *P* < 0.01, ^***^ *P* < 0.001, *vs.* NC+MC; ^^^ *P* < 0.05, ^^^^ *P* < 0.01, ^^^^^ *P* < 0.001, *vs.* circ+MC).

The viabilities of H1395 cells (**Figure 6A**) and A549 cells (**Figure 6B**) were detected after cell cultured for 24 and 48 h, and we discovered that CircCHST15 did not affect the cell viability, which was enhanced by miR-155-5p mimic (*P* < 0.05) and reduced by miR-194-5p mimic (*P* < 0.05) as compared with the NC+MC group. Mechanically, the cell proliferation of H1395 cells (**Figures 6C, D**) and A549 cells (**Figures 6E, F**) was assessed, consistent with cell viability, the cell proliferation was increased in the NC+miR-155-5p group (*P* < 0.001) and the circ+miR-155-5p group (*P* < 0.001) when compared with the NC+MC group and circ+MC group, respectively. However, the proliferation was inhibited in the NC+miR-194-5p group (*P* < 0.001) and in the circ+miR-194-5p group (*P* < 0.001) as compared with the NC+MC group and circ+MC group, respectively.

### CircCHST15 Regulated the Tumor Growth, T Cell Subtype, and the Secretion of Related-Factors in the Mouse Tumor Tissues

**Figure 7:**
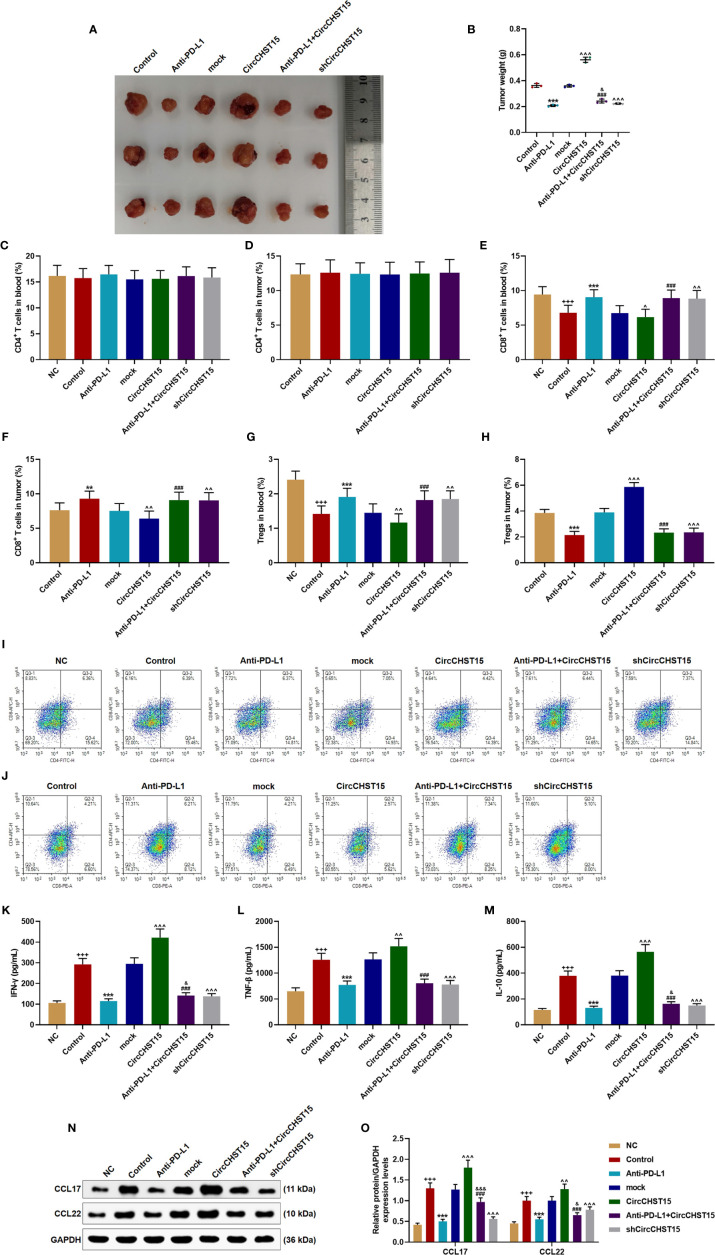
CircCHST15 regulated the tumor growth, the T cell subtype, and the secretion of related-factors in mouse tumor tissues. **(A)** The solid tumors were shown. **(B)** The weight of a solid tumor was calculated. **(C, D)** The level of CD4^+^ T cells in the blood **(C)** and tumor supernatant **(D)** of mice was quantified by the flow cytometer. **(E, F)** The level of CD8^+^ T cells in the blood **(E)** and tumor supernatant **(F)** of mice was quantified by the flow cytometer. **(G, H)** The level of Tregs in the blood **(G)** and tumor supernatant **(H)** of mice was calculated based on the formula: Tregs^+^ (%) = [(CD3^+^ + CD4^+^ + CD25^+^ + total cell number)/CD3^+^ + total cells number] × 100%. **(I, J)** The proportion of immune cell subsets in peripheral blood and tumor was measured by flow cytometry. **(K–M)** The levels of IFN-γ **(K)**, TNF-β **(L)**, and IL-10 **(M)** in the blood of mice were detected by ELISA. **(N, O)** The expressions of CCL17 and CCL22 in the mouse tumor tissues were detected by Western blot, GAPDH was used as an internal control. (^+++^ *P* < 0.001, *vs.* NC; **P < 0.01, ***P < 0.001, *vs.* Control; ^^^ *P* < 0.05, ^^^^ *P* < 0.01, ^^^^^ *P* < 0.001, *vs.* mock; ^###^ *P* < 0.001, *vs.* CircCHST15; ^&^ *P* < 0.05, ^&&&^ *P* < 0.001, *vs.* Anti-PD-L1).

After the establishment of a new lung cancer model in mice and the collection of tumor tissues (**Figure 7A**), the tumor weight (**Figure 7B**) was decreased by PD-L1 inhibitor (Anti-PD-L1) (*P* < 0.001) and shCircCHST15 (*P* < 0.001) when compared with the Control group and the mock group, respectively, but the tumor weight was increased by CircCHST15 (*P* < 0.001) when compared with the mock group. In the Anti-PD-L1+CircCHST15, the effects of CircCHST15 and Anti-PD-L1 on tumor weight were weakened by each other (*P* < 0.05) when compared with the Anti-PD-L1 group and the CircCHST15. As for the T cell subtype (**Figures 7C–H**), consistent with the results in **Figure 2**, the CD4^+^ T cells in the blood (**Figures 7C, I, J**) and tumor (**Figures 7D, I, J**) among these group showed no changes, but the CD8^+^ T cells and Tregs in the blood (**Figures 7E, G, I, J**) were decreased in the Control group (*P* < 0.05) and in the CircCHST15 group (*P* < 0.05), and increased by Anti-PD-L1 (*P* < 0.001) and shCircCHST15 (*P* < 0.01). However, in the Anti-PD-L1+CircCHST15, the effects of CircCHST15 on CD8^+^ T cells and Tregs were attenuated by Anti-PD-L1 (*P* < 0.001) when compared with the CircCHST15. The CD8^+^ T cells in the tumors (**Figures 7F, I, J**) showed the same tendency with CD8^+^ T cells in the blood among all the groups, while the Tregs in the tumors appeared the opposite tendency to Tregs in the blood among all the groups. Also, as shown in **Figures 7K–O**, the expressions of related-factors (IFN-γ, TNF-β, IL-10, CCL17, CCL22) were up-regulated in the Control group (*P* < 0.001) and the CircCHST15 group (*P* < 0.001), but down-regulated by Anti-PD-L1 (*P* < 0.001) and shCircCHST15 (*P* < 0.001). However, in the Anti-PD-L1+CircCHST15, the effect of CircCHST15 on the expression of TNF-β was attenuated by Anti-PD-L1 (*P* < 0.001) compared with the CircCHST15, moreover, the effects of CircCHST15 and Anti-PD-L1 on the expressions of IFN-γ, IL-10, CCL17, and CCL22 were attenuated by each other.

### CircCHST15 Regulated the Expressions of PD-L1, Ki67, and PCNA in the Mouse Tumor Tissues

**Figure 8:**
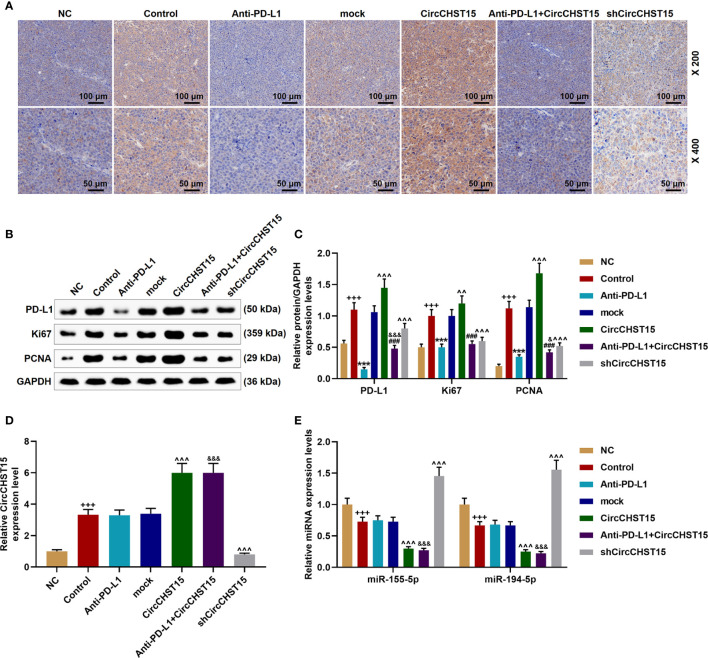
CircCHST15 regulated the expressions of PD-L1, Ki67, PCNA, miR-155-5p, and miR-194-5p in the mouse tumor tissues. **(A)** The expression of PD-L1 in the mice tumor tissues was detected by immunohistochemical assay. **(B, C)** The levels of PD-L1, Ki67, and PCNA in the tumor tissues were quantified by Western blot, GAPDH was used as an internal control. **(D)** The expression of CircCHST15 in the mouse tumor tissues was detected by RT-qPCR, GAPDH was used as an internal control. **(E)** The expressions of miR-155-5p and miR-194-5p in the mouse tumor tissues were detected by RT-qPCR, GAPDH was used as an internal control. (^+++^ *P* < 0.001, *vs.* NC; ^***^ *P* < 0.001, *vs.* Control; ^^^^^ *P* < 0.001, *vs.* mock; ^###^ *P* < 0.001, *vs.* CircCHST15; ^&^ *P* < 0.05, ^&&&^ *P* < 0.001, *vs.* Anti-PD-L1).

Finally, the expressions of PD-L1, CircCHST15, miR-155-5p, miR-194-5p, and proliferation-related factors (Ki67 and PCNA) in the tumor tissues were determined (**Figure 8**). As shown in **Figures 8A–C**, it could be observed that the expressions of PD-L1, Ki67, and PCNA were up-regulated in the Control group (*P* < 0.001) when compared with the NC group, but when compared with the Control group, these expressions were down-regulated by Anti-PD-L1 (*P* < 0.001), moreover, compared with the mock group, the above expressions were up-regulated by CircCHST15 (*P* < 0.001) and down-regulated by shCircCHST15 (*P* < 0.001). However, in the Anti-PD-L1+CircCHST15 group, the effect of CircCHST15 on the Ki67 expression was attenuated by Anti-PD-L1 (*P* < 0.001) compared with the CircCHST15 group, and the effects of CircCHST15 and Anti-PD-L1 on the expressions of PD-L1 and PCNA were weakened by each other when compared with the CircCHST15 (*P* < 0.001) and Anti-PD-L1 group (*P* < 0.05). As shown in **Figure 8D**, the expression of CircCHST15 was up-regulated in the Control group (*P* < 0.001) compared with the NC group, while in the Anti-PD-L1 did not affect the expression of CircCHST15. The expressions of miR-155-5p and miR-194-5p (**Figure 8E**) were found down-regulated in the Control group (*P* < 0.001) when compared with the NC group, similarly Anti-PD-L1 did not affect the expressions of miR-155-5p and miR-194-5p, which, however, were down-regulated by CircCHST15 (*P* < 0.001) and up-regulated by shCircCHST15 (*P* < 0.001) when compared with the mock group.

## Discussion

CircRNAs were abnormally expressed in different cancers (9, 11). In lung cancer, studies found that the expressions of hsa_circ_0072083, CircZNF124, and CircABCB10 are up-regulated (12, 14, 15) but that of hsa_circ_11780, CircPTK2, and hsa_circ_0078767 are down-regulated (13, 17, 18). This study discovered the up-regulation of CircCHST15 in lung cancer tissues and cells, which was similar to the findings of Liu et al. (16). We also established a lung cancer model (subcutaneous-xenotransplant) in mice, and found for the first time that down-regulated CircCHST15 inhibited the tumor growth, suggesting that CircCHST15 can act as an oncogene in lung cancer. Subsequently, our findings further showed that down-regulated CircCHST15 regulated distribution of T cell subtypes and inhibited the expression of PD-L1 in the tumor tissues, thus, we speculated that the effect of CircCHST15 on lung cancer might be associated with the immune system, as PD-L1 has critical functions in the immune response and immune escape in different cancers (19–22).

It is widely acknowledged that circRNAs can sponge some miRNAs to affect the functions of miRNAs in various biological activities (9, 11). Hsa_circ_0052112 sponges miR-125-5p to promote breast cancer cell migration (23); has_circ_0078710 participates in the progression of liver cancer by sponging miR-31 (24); hsa_circ_0012919 sponges miR-125a-3p to promote the progression of DNA methylation of CD11a in T cells (25). In this study, we found for the first time that miR-155-5p and miR-194-5p could be sponged by CircCHST15 in lung cancer cells. The expression of miR-155-5p is associated with the progression of lung cancer (26, 27). Previously, studies found that miR-155-5p and miR-194-5p regulate the metastasis, autophagy, apoptosis, drug resistance in cancers such as in cervical cancer, ovarian cancer, and breast cancer (28–33). In this study, the viability and proliferation of lung cancer cells were found to be promoted by miR-155-5p and inhibited by miR-194-5p. The target relationships between miR-155-5p with PD-L1 and between miR-194-5p with PD-L1 were further verified in this study. Furthermore, the observation that CircCHST15 did not affect the viability and proliferation of lung cancer cells further encouraged us to further focus on exploring the specific effect of CircCHST15 on immune escape in the development of lung cancer.

To examine the effect of CircCHST15 on immune escape mediated by regulating PD-L1, a lung cancer model (subcutaneous-xenotransplant) was established with the inhibitor of PD-L1 in mice. We found that PD-L1 inhibitor not only inhibited the growth of the tumor, but also reversed the effect of CircCHST15 on promoting tumor growth. Antibody blockade of PD-L1 can activate an anti-tumor immune response leading to durable remissions in a set of cancer patients (34). Anti-PD-1/PD-L1 therapy has generated significant clinical benefits in patients with non-small cell lung cancer (NSCLC) (35). High expression of PD-L1 in lung cancer may contribute to poor prognosis and tumor cells immune escape through suppressing tumor infiltrating dendritic cells maturation (36). EGFR-TKI resistance promotes immune escape in lung cancer *via* up-regulating PD-L1 expression (37). Autochthonous EGFR-driven lung tumors inhibit antitumor immunity by activating the PD-1/PD-L1 pathway to suppress T-cell function and increase levels of proinflammatory cytokines (38). During the progression of immune escape in cancers, the up-regulated PD-L1 regulates the expression of PD-L on the surface of T cells, which has strong immune functions, and then the binding of PD-L1 to PD-L further inhibits T cell functions (39). T cells are composed of CD4^+^ T cells and CD8^+^ T cells, which play different roles in immune responses (40). In this study, the proportion of CD4^+^ T cells remained stable in model mice, while that of CD8^+^ T cells was increased by PD-L1 inhibitor and inhibited by CircCHST15 in the mouse tumor, and PD-L1 inhibitor reversed the effect of CircCHST15. This indicated that the effect of CircCHST15 on promoting immune escape was mediated by suppressing the normal functions of CD8^+^ T cells. It has been reported that exosomal PD-L1 promoted tumor growth through immune escape in non-small cell lung cancer *via* inhibit CD8^+^ T-cell activity and induce the apoptosis of CD8^+^ T cells (41). Additionally, these results were supported by the expressions of key factors (IFN-γ, TNF-β, IL-10, CCL17, and CCL22) specific to the immune responses mediated by CD8^+^ T cells. Noticeably, we further confirmed that after the overexpression CircCHST15, the expressions of miR-155-5p and miR-194-5p were down-regulated and PD-L1 expression was up-regulated in the tumor tissues.

To conclude, our research discovered that CircCHST15 sponged miR-155-5p and miR-194-5p to promote the PD-L1-mediated immune escape in lung cancer.

## Data Availability Statement

The original contributions presented in the study are included in the article/supplementary material. Further inquiries can be directed to the corresponding author.

## Ethics Statement

The animal study was reviewed and approved by Second Affiliated Hospital of Chongqing Medical University.

## Author Contributions

JY provided substantial contributions to the conception and design. YJ, BW, SY, KD, YjL, YhL, and BZ acquired, analyzed, and interpreted the data. JY drafted the article or critically revising it for important intellectual content. All authors agreed to be accountable for all aspects of the work in ensuring that questions related to the accuracy or integrity of the work are appropriately investigated and resolved. All authors contributed to the article and approved the submitted version.

## Conflict of Interest

The authors declare that the research was conducted in the absence of any commercial or financial relationships that could be construed as a potential conflict of interest.
